# Molecularly Imprinted Polymers for Removal of Metal Ions: An Alternative Treatment Method

**DOI:** 10.3390/biomimetics3040038

**Published:** 2018-11-30

**Authors:** Özgecan Erdem, Yeşeren Saylan, Müge Andaç, Adil Denizli

**Affiliations:** 1 Department of Biology, Hacettepe University, 06800 Ankara, Turkey; ozgecanerdem@hacettepe.edu.tr; 2 Department of Chemistry, Hacettepe University, 06800 Ankara, Turkey; yeseren@hacettepe.edu.tr; 3 Department of Environmental Engineering, Hacettepe University, 06800 Ankara, Turkey; andac@hacettepe.edu.tr

**Keywords:** ion imprinting, molecularly imprinted polymers, metal pollution, metal removal

## Abstract

Aquatic and terrestrial environment and human health have been seriously threatened with the release of metal-containing wastewater by the rapid growth in the industry. There are various methods which have been used for removal of ions from the environment, such as membrane filtration, ion exchange, membrane assisted liquid extraction and adsorption. As a sort of special innovation, a polymerization technique, namely molecular imprinting is carried out by specific identification for the target by mixing it with a functional monomer. After the polymerization occurred, the target ion can be removed with suitable methods. At the end of this process, specific cavities, namely binding sites, are able to recognize target ions selectively. However, the selectivity of the molecularly imprinted polymer is variable not only because of the type of ligand but also charge, size coordination number, and geometry of the target ion. In this review, metal ion-imprinted polymeric materials that can be applied for metal ion removal from different sources are discussed and exemplified briefly with different metal ions.

## 1. Introduction

Pollution of water is a significant worldwide threat. Various kinds of chemical pollutants have been discharged to the environmental water by different industries and agricultural applications [1]. Modern industries like battery manufacturing, mining, metal plating, and pesticides facilities release heavy metals in the environment. Heavy metals generally have a density higher than 5 g/cm^3^. Contamination of water with heavy metal ions affects the ecosystem seriously and this creates important problems [2]. The existence of hazardous metal ions in the aquatic environment has become a problem because of their detrimental effects on human health and other organisms [3]. The exposure of organisms to even very low concentrations of metals is extremely toxic and can greatly impair biological processes. This emerges from the ability that metal ions have to accumulate in nature and food chains [4].

There are different methods used for the removal of metal ions from water and wastewater such as membrane processes, chemical precipitation, extraction, ion exchange and adsorption [5]. Molecular imprinting is a novel polymerization technology to constitute selective recognition sites for a target in a polymeric matrix [6]. The template, functional monomers, cross-linkers and initiators are necessary for the polymerization. During the polymerization, the template molecule forms a complex with the functional monomer. The monomer–template complex is copolymerized in the presence of a cross-linker which constitutes a three-dimensional polymeric matrix around the template molecule. After polymerization, the template is removed and a target specific recognition cavity is formed in the polymeric matrix.

The form of the molecularly imprinted polymer can be a micro-/nanosized particle, hydrogel, cryogel, or monolith with template specific binding sites [7]. The binding affinity between the template and functional monomer is one of the main important parameters for specific template recognition. Thus, the complexation is the crucial step to have the optimum combination of the template and functional monomer [8]. The cross-linkers make the polymer chain bind to another. They act as critical functions to determine the features of polymers. The main function of the cross-linker is to produce a stable polymeric matrix and also comprise recognition sites [9]. The initiators are chemicals that react with a monomer to produce a compound that is able to link with a large number of other monomers into a polymeric matrix. Initiators should be selected according to the polymerization type and template molecule [10]. The solvent type can cause collapse of polymer chains in solution to form a hard sphere or swelling in order to maximize the number of polymer–fluid contacts. The solvent acts as a pore maker which has an impact on the morphology and porosity of the polymeric matrix [11]. 

**Figure 1:**
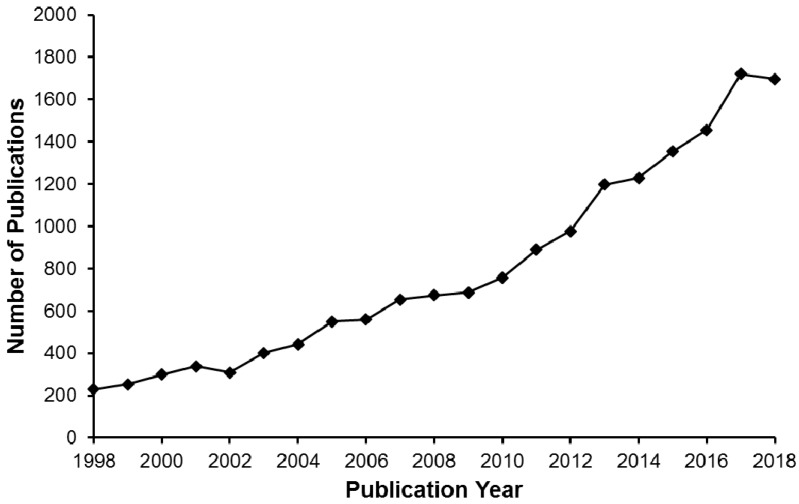
The number of publications for metal ion-imprinted polymers.

Molecularly imprinted polymers can be prepared for any molecule depending on the application area. More than 10,000 molecules and biological structures like metal ions, hormones, proteins and cells have been imprinted successfully [12,13,14,15]. Metal ion imprinting, based on molecular imprinting technology, is used for preparing materials that can recognize metal ions. These ion-imprinted materials can be used both for detection and removal of metals. The polymers are prepared in the presence of the ions. When the template is removed, specific cavities show high selectivity to the desired metal ion [16,17]. Studies on metal ion-imprinted polymers have been increasing over the years as shown in Figure 1. In this statistics, “imprint” and “metal” were selected as keywords to classify the number of publications in the Science Direct database [18]. According to the results, the total publication numbers are calculated as 16,674 since 1998. It can be seen that the number of publications for metal ion-imprinted polymers is increasing every year.

Here, molecularly imprinted polymer applications for different metal ions are reviewed and various examples with metal ions discussed according to their contributions to the literature. 

## 2. Removal of Metal Ions with Imprinted Materials

### 2.1. Mercury

Mercury is a metal that occurs naturally and is released primarily through geothermal activity on Earth [19]. The toxicity of mercury in soil is based mainly on its chemical properties. Methylmercury, the most poisonous form of mercury, has a high affinity for the sulfhydryl ligands in amino acids, which reduces changes in protein structures and leads to a loss of function. Due to the persistent mercury accumulation in the aquatic environment, mercury contamination is among the most studied of all environmental pollutants [20]. There are several techniques for mercury removal from water such as precipitation/co-precipitation, ion exchange, membrane filtration, bioremediation and adsorption [4].

Zhang et al. [21] synthesized magnetic mercury (II)-imprinted polymeric nanoparticles using allylthiourea as a functional monomer. They measured magnetization of the magnetic mercury (II)-imprinted polymeric nanoparticles as 15.94 emu/g. The adsorption capacity of the magnetic mercury (II)-imprinted polymeric nanoparticles towards mercury ions was obtained as 78.3 mg/g, twice of magnetic nonimprinted polymeric nanoparticles (39.5 mg/g). They also calculated relative selectivity coefficients of the magnetic mercury (II)-imprinted polymeric nanoparticles for other competitor ions, respectively. They applied the magnetic mercury(II)-imprinted polymeric nanoparticles in real samples and measured a removal rate of more than 99% of that below the United States Environmental Protection Agency mercury limits for wastewater. 

Mergola et al. [22] published a study on mercury (II)-imprinted polymeric sorbents by different approaches using diphenylcarbazone as a chelating agent for mercury ions removal from solutions. The first and second mercury (II)-imprinted polymeric sorbents were prepared in the absence and presence of diphenylcarbazone, respectively; and the third mercury (II)-imprinted polymeric sorbent was also prepared to estimate the contribution of characteristic properties on adsorption capacity. They demonstrated the pertinency of the mercury (II)-imprinted polymeric sorbents through batch experiments using mercury (II)-spiked drinking water and observed that the best removal efficiency was of almost 80% for the second mercury (II)-imprinted polymeric sorbent.

**Figure 2:**
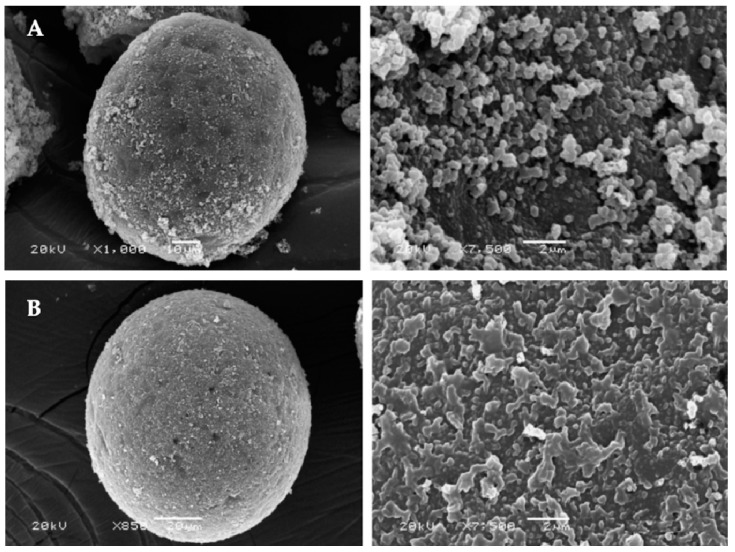
Scanning electron microscope images of (**A**) mercury (II)-imprinted polymeric beads and (**B**) nonimprinted polymeric beads. Republished with permission of Elsevier, from [23]; permission conveyed through Copyright Clearance Center, Inc.

In another study, Andaç et al. [23] prepared ion-imprinted polymeric beads for mercury (II) removal from human serum. They used an amino acid as the complexing monomer to obtain mercury (II)-imprinted polymeric beads. After that, they characterized these beads by several methods (Figure 2). The surface area was measured as 59.04 m^2^/g with a size range of 63–140 µm. They also observed a maximum adsorption capacity of 0.45 mg/g. According to the results, they showed that the mercury (II)-imprinted polymeric beads could be used several times without reducing their adsorption capacities. 

Xu et al. [24] synthesized a functional monomer, 3-isocyanatopropyl triethoxysilane bearing thymine bases, and then prepared solid-phase extraction sorbents for mercury (II) pre-concentration in water samples. According to the results, they obtained satisfactory recoveries ranging from 95.2 to 116.3% and also pre-concentration factor and limit of detection values were achieved of 200 and 0.03 μg/L. Furthermore, the reusability tests showed that the solid-phase extraction sorbents could be used even after five adsorption-desorption cycles without a noteworthy decrease in adsorption capacity.

### 2.2. Copper

Copper is also a common toxic ion and excessive amounts of copper are dangerous to the environment and organisms. Copper pollution affects the ecosystem of urban areas. In addition, copper commonly affects the chemoreception and chemosensory abilities of aquatic animals which underlie key interactions including finding prey, avoiding predators, and detecting conspecifics [25]. Therefore, it will give rise to vital troubles if copper ions leak into the aquatic system without treatment. Several techniques were reported for removal of copper ions for environmental analysis [26].

A multi-ion imprinting method was proposed for pre-concentration and removal of different ions (copper (II), mercury (II), cadmium (II) and nickel (II)) by Fu et al. [27]. The physical and chemical properties of multi-ion imprinted polymeric sorbents were characterized by various analyses. They showed that the multi-ion imprinted polymeric sorbents have high binding capacities and rapid dynamics and also high selectivity with selective coefficients of 6.8–16.9 toward the competitor ions. They employed solid-phase extraction sorbents for pre-concentration of ions showing high detectability of up to 6.0–22.5 ng/L in seawater samples.

Ren et al. [26] prepared copper (II)-imprinted polymeric material by a sol–gel method for copper (II) ions removal from aqueous solution. After several characterization experiments, they showed that the three-dimensional network was formed and functional monomer was successfully cross-linked into the copper (II)-imprinted polymeric material. They also reported that the maximum adsorption capacity (39.82 mg/g) of the copper (II)-imprinted polymeric material was higher than that of the nonimprinted polymeric material. In addition, they demonstrated that the copper (II)-imprinted polymeric material showed high selectivity toward other metal ions (lead (II), nickel (II), cadmium (II) and cobalt (II)) and could be reused many times without any adsorption capacity loss.

Kong et al. [28] developed copper (II)-imprinted polymeric materials employing graphene oxide, acrylamide, and ethylene glycol dimethacrylate in the presence of copper (II) ions. After the characterization analysis, they investigated optimum condition to maximum copper (II) adsorption. They showed that copper (II)-imprinted polymeric materials had a high imprinting factor and the maximum adsorption capacity was 132.77 mg/g and also the copper (II)-imprinted polymeric materials showed an extensive application for recovery of copper (II) ions from aqueous solutions. 

### 2.3. Lead

Lead arises commonly associated with zinc, copper and silver ores in the environment. It is generally employed for several industrial applications such as paint, cables, pesticides and pipelines and the main anthropogenic input is through the fossil fuel of combustion engines [29]. Among other metals, lead is one of the most dangerous pollutants of the environment and lead pollution in the water, air and agricultural soil is an ecological problem because of its negative impact on human health and the environment [30].

Mishra et al. [31] reported a study about lead (II)-imprinted and carbon nanofibers-grafted polymeric beads. They first synthesized allylthiourea based polymeric beads and then mixed with carbon nanofibers during the polymerization before the curing step. They obtained a high adsorption capacity (47 mg/g) of lead with lead (II)-imprinted and carbon nanofiber-grafted polymeric beads in aqueous solution and also high selectivity coefficients toward competitive ions. They regenerated lead (II)-imprinted and carbon nanofiber-grafted polymeric beads by acid treatment and used them in five adsorption/regeneration cycles. They claimed that the lead (II)-imprinted and carbon nanofiber-grafted polymeric beads can be applied for the development of similar polymeric materials for removal of other toxic metal ions present in industrial effluents. 

**Figure 3:**
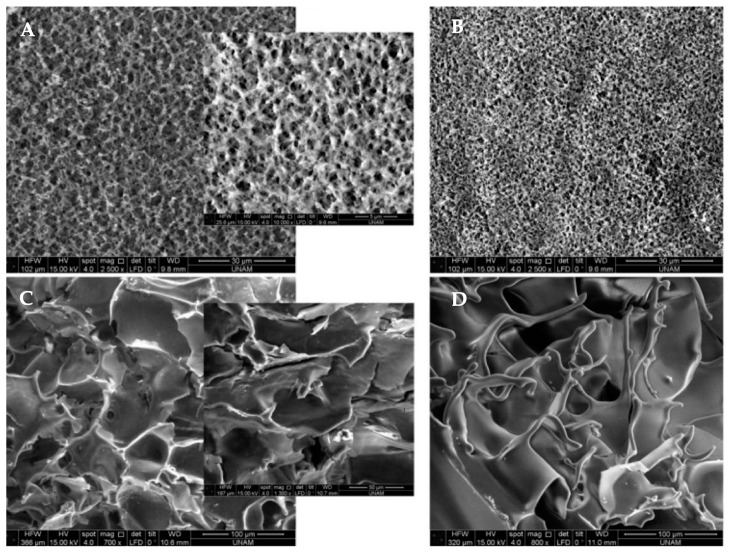
Scanning electron microscope images of (**A**) cadmium (II)-imprinted, (**B**) nonimprinted, (**C**) lead (II)-imprinted and (**D**) nonimprinted polymeric cryogels. Republished with permission of John Wiley & Sons, Inc., from [5]; permission conveyed through Copyright Clearance Center, Inc.

Denizli and his research group synthesized amino acid-based lead (II)-imprinted polymeric cryogels [5]. They calculated the maximum adsorption capacities to be around 42, 86, and 123 mg/g for lead (II)-imprinted polymeric cryogels. They observed that the adsorption was fast which was associated with supermacroporous and interconnected channels of cryogels (Figure 3). They also performed mathematical calculations and found that Langmuir and pseudo-second order kinetic models were fitted to experimental results. They finally compared the adsorption capacities with multi-ion synthetic wastewater to demonstrate that the lead (II)-imprinted polymeric cryogels can be applied to metal recycling. 

**Figure 4:**
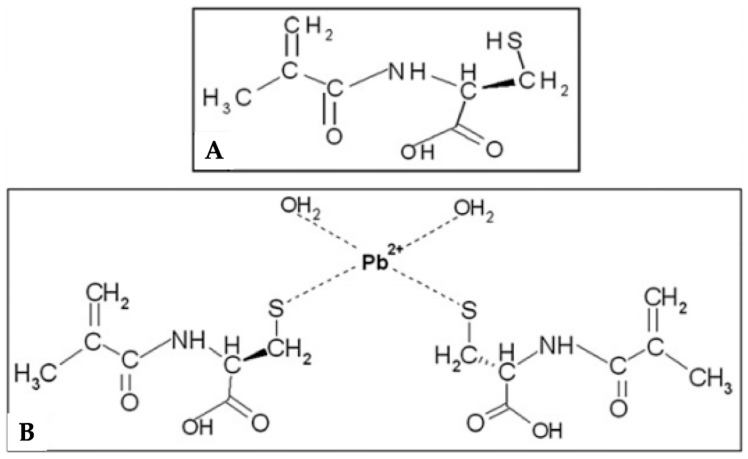
(**A**) Chemical structure of the *N*-methacryloyl-(l)-cysteine monomer and (**B**) pre-complex of template and monomer. Republished with permission of Elsevier, from [32]; permission conveyed through Copyright Clearance Center, Inc.

Esen et al. [32] prepared a lead (II)-imprinted polymeric particles using an amino acid-based monomer (Figure 4). The imprinted particles were used as a solid-phase extraction adsorbent. After the removal of lead (II) ion, high selective cavities occurred. According to the characterization results, the size of the lead (II)-imprinted polymeric particles was from 50 to 200 μm with a rough surface and macropores in bulk structure, and presented low adsorption capacity (2.01 mg/g). Selectivity studies were performed with different ions (cadmium (II), nickel (II) and copper (II)). Moreover, the reusability of the lead (II)-imprinted polymeric particles were tested for several times and no significant loss in adsorption capacity was observed.

A highly selective lead (II)-imprinted polymer was prepared by Cai et al. [33] based on the synergy of two different functional monomers; methacrylic acid and vinylpyridine with the purpose of solid-phase extraction of lead (II) in water samples. The prepared polymer showed high selectivity to lead (II) with a high coefficient above 30. The results showed that a good linearity (*R*^2^ = 0.9998) was obtained in the range of 0.2−50 μg/L. The limit of detection and quantification values was calculated as 0.06 and 0.19 μg/L, respectively. Real sample studies were also performed with lake and tap water, and according to the results, recovery values varying from 95.5 to 104.6% were obtained.

### 2.4. Cadmium

Cadmium is a poisonous and cancer-causing metal that can happen as a food contaminant and worldwide pollutant. Long-term occupational exposure to high cadmium concentrations may cause lung cancer, kidney and bone damages and hematuria [34]. Variance exists on the maximum advisable concentration limit of several metal ions in water. The content for cadmium ions is less than 1 μg/L in nonpolluted fresh water, while the World Health Organization limit for drinking water is 10 μg/L [35]. 

**Figure 5:**
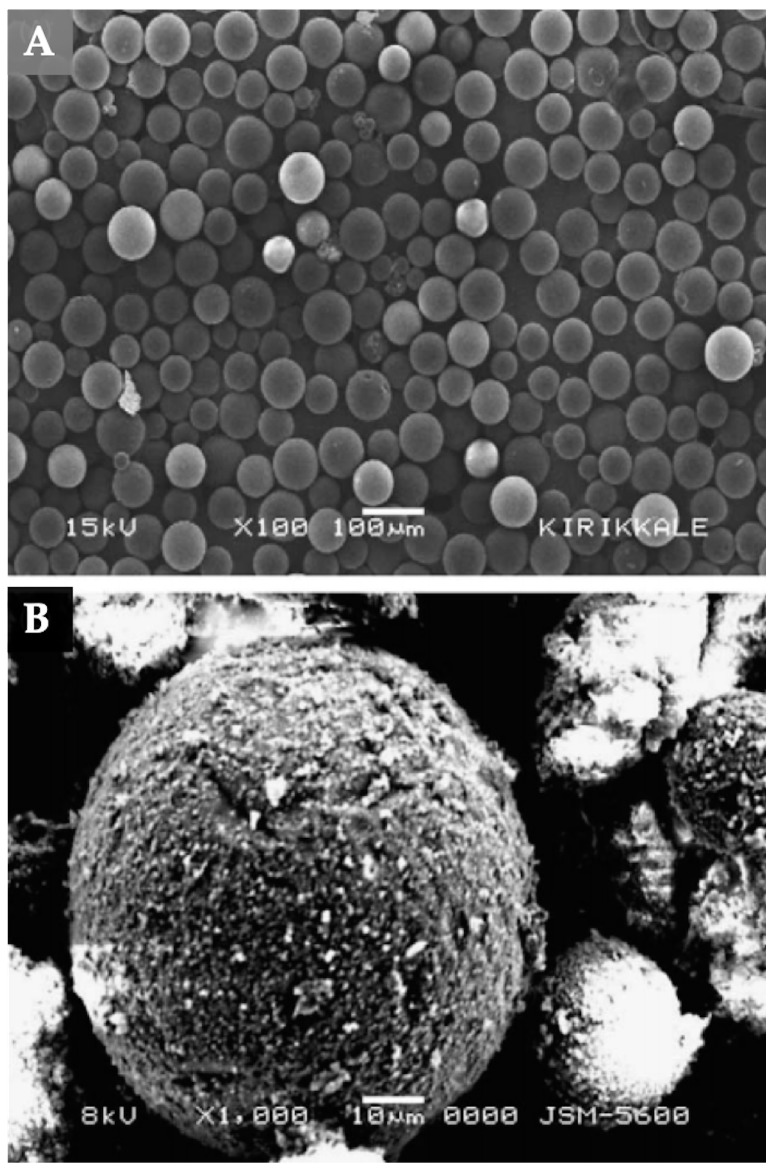
Scanning electron microscopy images of cadmium (II)-imprinted polymeric beads. (**A**) surface and (**B**) internal structure. Republished with permission of Elsevier, from [36]; permission conveyed through Copyright Clearance Center, Inc..

Cadmium (II)-imprinted polymeric beads were prepared for removal of cadmium ions from cadmium-overdosed human plasma by Andaç et al. [36]. They measured the swelling ratio as 78% and specific surface area of the cadmium (II)-imprinted polymeric beads as 19.4 m^2^/g with a size range of 63–140 µm in diameter (Figure 5). They also calculated the adsorption capacity as 32.5 µmol/g. In addition, they obtained higher relative selectivity coefficients of imprinted beads than nonimprinted beads and showed that the cadmium (II)-imprinted polymeric beads could be used many times without decreasing their adsorption capacities significantly. 

Rahangdale et al. [16] reported a study about molecularly dual imprinted polymeric material for the removal of cadmium by suspension polymerization. Scanning electron microscopy, swelling study and Fourier transform infrared spectroscopy indicated successful polymerization of the dual-ion imprinted polymeric material. Based on kinetic studies, they calculated an adsorption capacity of 38.46 mg/g under the optimum condition and also observed the adsorption capacity of the molecularly dual-ion imprinted polymeric material increased with contact time and reached the equilibrium at 90 min. Adsorption isotherm results well fitted into the Langmuir model with a high correlation coefficient (0.994). 

Cadmium (II)-imprinted polymeric materials were also prepared by Li et al. [35]. They prepared a nonimprinted polymeric material and compared the adsorption capacities of the cadmium (II)-imprinted and nonimprinted polymeric materials. Kinetics analyses showed that the equilibrium was completed in 8 min and the adsorption was fitted with pseudo-second order kinetic model. In addition, they observed that cadmium (II) adsorbed and removed easily from the sorbent and the cadmium (II)-imprinted polymeric material exhibited good stability and reusability. They verified the accuracy of the cadmium (II)-imprinted polymeric material preparation method by the standard reference material and applied for cadmium (II) determination in different types of water samples.

### 2.5. Chromium

Chromium is also extensively used in different industries like leather tanning, photography and metal cleaning. Industrial wastewater containing heavy metal ions discharged to the environment and their accumulation is an important source of water pollution [37]. Chromium (III) is stable and less toxic and also considered an essential element for many organisms. Chromium (VI) is more toxic, mutagenic and carcinogenic. According to The United States Environmental Protection Agency the maximum contaminant level for chromium (VI) in surface water is 0.1 mg/L [38]. Over the maximum level the ion is hazardous and must be removed from the environment. 

**Figure 6:**
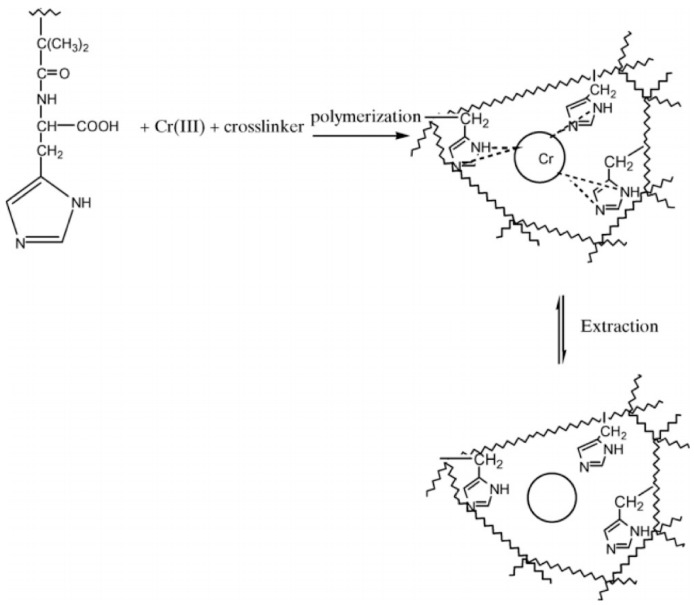
Preparation of chromium (III)-imprinted polymeric adsorbent. Republished with permission of Elsevier, from [39]; permission conveyed through Copyright Clearance Center, Inc.

A molecularly imprinted polymeric adsorbent for chromium (III) analysis was prepared by Birlik et al. [39]. First, the chromium (III)-methacryloyl histidine pre-complex was prepared; after that, chromium (III)-imprinted ethylene glycol dimethacrylate-*N*-methacryloyl-(l)-histidine was polymerized as seen in Figure 6. The maximum adsorption of chromium (III) was found to be 69.28 mg/g. Sorption studies of cobalt (II), nickel (II), and chromium (VI) ions were also performed in order to determine the selectivity of the chromium (III)-imprinted polymeric adsorbent. The results showed that the adsorbed amount of chromium (III) on the polymeric adsorbent was higher than that of other metal ions. 

Another study about chromium (VI)-imprinted polymeric nanoparticles was conducted by Uygun et al. [40]. These researchers prepared chromium (VI)-imprinted polymeric nanoparticles to remove chromium (VI) from wastewater. Chromium (VI) ions were mixed with *N*-methacryloylamido histidine in order to prepare a pre-complex and then chromium (VI)-imprinted polymeric nanoparticles were synthesized using the surfactant-free emulsion polymerization. The particle size was measured to be 155.3 nm. Selectivity studies were performed with chromium (III) ion and according to the results, chromium (VI)-imprinted polymeric nanoparticles showed high affinity to chromium (VI) ion. The chromium (VI)-imprinted polymeric nanoparticles were used several times without decreasing their chromium (VI) adsorption capacities. 

### 2.6. Nickel

Nickel is a silvery-white transition metal that takes on a high polish. The toxicity of nickel depends on the way of its exposure and the solubility of the compound like other metals [41].

Zhou et al. [42] prepared a nickel (II)-imprinted polymeric material using the bulk polymerization method. Different monomers, cross-linking agents, solvents, and molar ratios of templates were investigated to find out the highest adsorption capacity. After several characterization studies, the maximum adsorption capacity of the nickel (II)-imprinted polymeric material was found to be 86.3 mg/g at pH 7.0 and the initial nickel (II) concentration was 500 mg/L. The selectivity coefficients for all nickel-interfering ions was found higher than 1.

**Figure 7:**
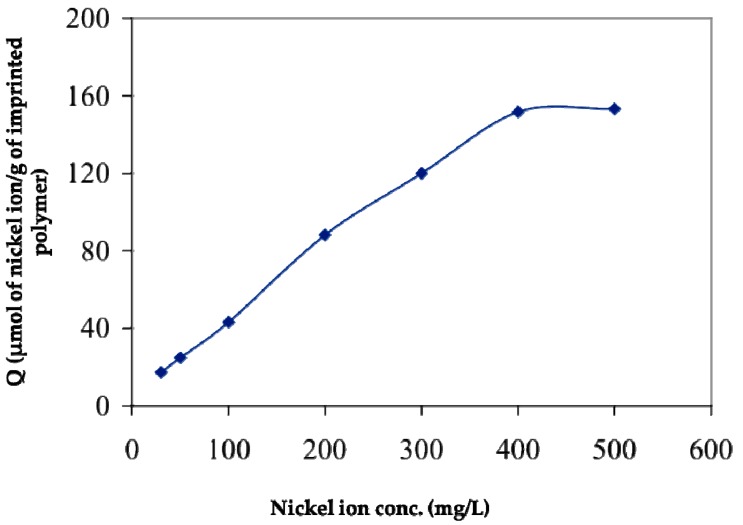
Effect of nickel (II) concentration on the amount of adsorbed nickel (II). Republished with permission of Elsevier, from [43]; permission conveyed through Copyright Clearance Center, Inc.

Ersöz et al. [43] prepared a solid-phase extraction polymeric column in order to separate and pre-concentrate nickel (II) from aqueous solution. After the removal of nickel (II) ions from the polymeric matrix, it was used for extraction of nickel (II) from aqueous solution. As shown in Figure 7, the amount of nickel (II) adsorbed per unit mass of the polymeric column increased with the initial concentration of the nickel (II). They reported that the pre-concentration procedure showed a linear calibration curve within the concentration range 0.3–25 ng/mL and the detection limit was 0.3 ng/mL for flame atomic absorption spectrometry. Seawater was used as a real sample in the study and according to the results, the nickel (II)-imprinted polymeric column showed excellent selectivity even in the presence of a complicated medium like seawater.

In a study conducted by Tamahkar et al. [44], nickel (II)-imprinted polymeric cryogels were prepared. Two different nickel (II)-imprinted polymeric cryogels were synthesized with different functional monomer/template complexing molar ratios. The maximum adsorption capacities of nickel (II)-imprinted polymeric cryogels were found to be 1.89 and 5.54 mg/g for the first and second nickel (II)-imprinted polymeric cryogels, respectively. Iron (III), copper (II) and zinc (II) were used as competitive metal ions and nickel (II)-imprinted polymeric cryogels showed high selectivity toward these ions. Moreover, nickel (II)-imprinted polymeric cryogels were utilized over and again without a decrease in the binding capacity.

### 2.7. Other Metals

In addition to the metal ions mentioned above, there are also other metals like manganese, aluminum, and cobalt which cause environmental pollution as well. These metals are briefly mentioned in this section. Manganese is a metal ion which is used in electrochemical, chemical, food and pharmaceutical applications. It is also used in ferrous metallurgy generally. Despite the fact that it is fundamental for human life, at levels exceeding 0.1 mg/L, the existence of manganese (II) in drinking water over the limits may cause accumulation and impact the nervous system [45].

Khajeh-Sanchooli et al. [46] prepared manganese (II)-imprinted polymeric materials with the purpose of determining and removing manganese (II) in water samples. The effects of the independent variables and their interactions were examined statistically. According to the results, the amount of polymer, the pH of the solution, and adsorption time were found statistically significant. Box-Behnken design techniques were used with response surface methodology for optimization of manganese (II) removal by the imprinted polymeric material. The optimum pH was 9.7, with an optimum amount of polymer of 44.4 mg and adsorption time of 19.1 min. The detection limit was observed at 0.6 μg/L under the optimized conditions. 

In a study conducted by Andaç et al. [47], aluminum (III)-imprinted polymeric beads were prepared by suspension polymerization for selective removal of ions from aqueous solutions. The specific surface area of the aluminum (III)- imprinted polymeric material was found to be 55.6 m^2^/g with a size range of 63–140 μm. Elemental analysis was also conducted and the results showed that the aluminum (III)-imprinted polymeric beads contained 640 μmol/g. The maximum adsorption capacity was 122.9 μmol/g. The aluminum (III)-imprinted polymeric beads can be used numerous times and the results showed that there is no significant decrease in their adsorption capacities.

A cobalt (II)-imprinted polymeric material was synthesized by Yuan et al. [48] in order to remove metal ion from the environment. The cobalt (II)-imprinted polymeric material showed an adsorption capacity on cobalt (II) as high as 175 mg/g with high selectivity over other metal ions. In addition, the selectivity coefficient of cobalt (II)-imprinted polymeric material toward nickel (*k* = 4.17) is higher than the nonimprinted polymer (*k* = 0.74). These finding suggest that this methodology will introduce new opportunities in the area of removing metal ions and radioactive nuclides.

**Figure 8:**
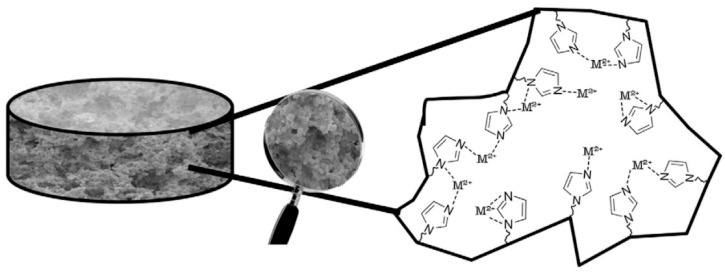
Scheme of the pre-complex formation between imidazole groups and metal ions. Republished with permission of Elsevier, from [49]; permission conveyed through Copyright Clearance Center, Inc.

Tekin et al. [49] prepared ion-imprinted cryogels with imidazole functional groups by two different methods (Figure 8). All cryogels were used in order to remove lead (II), cadmium (II), zinc (II) and copper (II) ions from aqueous solution. The surface area of the first cryogel was 39.7 m^2^/g, while the second one was 78.6 m^2^/g. The decrease of adsorption capacities of the cryogels were calculated as 38.5% for copper (II), 39.1% for lead (II), 66.9% for zinc (II) and 69.9% for cadmium (II). The maximum adsorption capacities of the cryogel were found for lead (II), cadmium (II), zinc (II) and copper (II) to be 7620, 5800, 4340 and 2540 µg/g, respectively. According to the results, ion-imprinted cryogels could be reused without a critical decrease in the adsorption capacity even after ten adsorption–desorption processes. All these studies have been summarized according to different parameters (monomers, cross-linkers, initiators and conditions of synthesis, etc.) in Table 1. 

## 3. Conclusions and Perspectives

Extraction and measurement of metal ions from the aqueous environment remains a serious problem because of their toxicity and cancer risk. Because of this reason, ion-imprinted polymeric materials have been developed further over the last two decades. Especially, they have gained great attention in many areas of science such as chemistry, physics, biology, biochemistry and biotechnology. The most important reason is owing to its selectivity and affinity to the target molecules [50]. Molecularly imprinted polymers are more robust, strong and resistant to physical factors (temperature, pH, organic solvents, etc.) than biological systems. They also offer a facile and cost-effective synthesis and can be stored for a long time [51]. All these advantages make molecularly imprinted polymers suitable to use in environmental pollution management. Molecularly imprinted polymers can be successfully prepared at the lab-scale. New methods have begun to be discovered with the scientific progress and some innovations should be done for using these materials in pilot-scale and large-scale applications. Using metal ion imprinted polymers in order to remove metal ions from the aquatic environment has begun to be employed and their application will continue to expand in the future.

## Figures and Tables

**Table 1:** Comparison of different studies for meal ion treatments.

Metal Ion	Monomer	Cross-linker	Initiator	Synthesis Conditions	Treatment Material	Adsorption Capacity	Reference
Mercury (II)	Allylthiourea	Ethylene glycol dimethacrylate	*N*,*N*-Azobisisobutyronitrile	65 °C, 12 h	Magnetic polymer	78.3 mg/g	[21]
Mercury (II)	4-Vinylpyridine	Ethylene glycol dimethacrylate	*N*,*N*-Azobisisobutyronitrile	65 °C, 24 h	Polymeric sorbent	70 mg/g	[22]
Mercury (II)	*N*-Methacryloyl-(l)-cysteine	Ethylene glycol dimethacrylate	Benzoyl peroxide	-	Polymeric bead	0.45 mg/g	[23]
Mercury (II)	3-Isocyanatopropyl triethoxysilane	Tetraethoxysilicane	Ammonia	60 °C, 6 h	Solid-phase extraction sorbent	2.8 mg/g	[24]
Copper (II)	Dithizone	Tetraethoxysilicane	Ammonia	25 °C, 12 h	Ion-imprinted polymeric sorbent	16.55 mg/g	[27]
Copper (II)	*N*-[3-(2-aminoethylamino) propyl] trimethoxysilane	Tetraethyl orthosilicate	Ammonia	60 °C, 24 h	Ion-imprinted polymer	39.82 mg/g	[26]
Copper (II)	Acrylamide	Ethylene glycol dimethacrylate	*N*,*N*-Azobisisobutyronitrile	65 °C, 8 h	Sandwich-like ion-imprinted polymer	132.77 mg/g	[28]
Lead (II)	Allylthiourea	Ethylene glycol dimethacrylate	*N*,*N*-Azobisisobutyronitrile	80 °C, 6 h	Carbon nanofiber ion-imprinted polymeric bead	47 mg/g	[31]
Lead (II)	*N*-Methacryloyl-(l)-cysteine	Methylenebisacrylamide	Ammonium persulfate	−12 °C, 24 h	Ion-imprinted cryogel	122.7 mg/g	[5]
Lead (II)	*N*-Methacryloyl-(l)-cysteine	Ethylene glycol dimethacrylate	Potassium persulfate	75 °C, 1 h	Ion-imprinted particle	2.01 mg/g	[32]
Lead (II)	Methacrylic acid, 4-vinyl pyridine	Ethylene glycol dimethacrylate	*N*,*N*-Azobisisobutyronitrile	60 °C, 8 h	Ion-imprinted polymer	8.35 mg/g	[33]
Cadmium (II)	*N*-Methacryloyl-(l)-cysteine	Ethylene glycol dimethacrylate	Benzoyl peroxide	90 °C, 2 h	Ion-imprinted polymer bead	32.5 µmol/g	[36]
Cadmium (II)	Chitosan	Epichlorohydrin	-	50 °C, 4 h	Ion dual imprinted polymer	38.46 mg/g	[16]
Cadmium (II)	Allyl thiourea	Ethylene glycol dimethacrylate	*N*,*N*-Azobisisobutyronitrile	333 K, 24 h	Ion-imprinted polymer	38.30 mg/g	[35]
Chromium (III)	2-Methacryloylamido histidine	Ethylene glycol dimethacrylate	*N*,*N*-Azobisisobutyronitrile	90 °C, 3 h	Ion-imprinted polymeric bead	69.28 mg/g.	[39]
Chromium (VI)	2-Methacryloylamido histidine	Ethylene glycol dimethacrylate	Potassium persulfate	70 °C, 3 h	Ion-imprinted nanoparticle	3830.58 mg/g	[40]
Nickel (II)	Methacrylic acid	Ethylene glycol dimethacrylate	*N*,*N*-Azobisisobutyronitrile	60 °C, 24 h	Ion-imprinted polymer	86.3 mg/g	[42]
Nickel (II)	2-Methacryloylamido histidine	Ethylene glycol dimethacrylate	*N*,*N*-Azobisisobutyronitrile	90 °C, 3 h	Solid-phase extraction polymeric column	160 µmol/g	[43]
Nickel (II)	2-Methacryloylamido histidine	Poly(ethylene glycol) diacrylate	Ammonium persulfate	−12 °C, 24 h	Ion-imprinted cryogel	5.54 mg/g	[44]
Manganese (II)	4-Vinylpyridine	Ethylene glycol dimethacrylate	*N*,*N*-Azobisisobutyronitrile	60 °C, 5 h	Ion-imprinted polymer	44.4 mg/g	[46]
Aluminum (III)	*N*-Methacryloyl-(l)-glutamic acid	Ethylene glycol dimethacrylate	Benzoyl peroxide	-	Ion-imprinted polymeric bead	122.9 μmol/g	[47]
Cobalt (II)	Glycylglycine	Glutaraldehyde	-	60 °C, 5 h	Ion-imprinted polymer	175 mg/g	[48]
Multi-ions	*N*-Vinylimidazole	Ethylene glycol dimethacrylate	*N*,*N*-Azobisisobutyronitrile	90 °C, 2 h	Ion-imprinted cryogel	7620–2540 μg/g	[49]
